# A pharmacological mouse model suggests a novel risk pathway for postpartum psychosis

**DOI:** 10.1016/j.psyneuen.2016.09.019

**Published:** 2016-12

**Authors:** Trevor Humby, Ellen S. Cross, Lauren Messer, Silvia Guerrero, William Davies

**Affiliations:** a School of Psychology, Cardiff University, Tower Building, 70, Park Place, Cardiff, CF10 3AT, UK; b Neuroscience and Mental Health Research Institute, Cardiff University, Hadyn Ellis Building, Maindy Road, Cardiff, CF24 4HQ, UK; c Medical Research Council Centre for Neuropsychiatric Genetics and Genomics and Division of Psychological Medicine and Clinical Neurosciences, Cardiff University, Hadyn Ellis Building, Maindy Road, Cardiff, CF24 4HQ, UK; d University of Barcelona, Gran Via de les Corts Catalanes, 585 08007 Barcelona, Spain

**Keywords:** 667-COUMATE, Elevated plus maze, Nephroblastoma-overexpressed, Startle response, Steroid sulfatase

## Abstract

Postpartum psychosis (PP) is a severe psychiatric disorder affecting a small proportion of new mothers shortly after childbirth. The molecular pathophysiology underlying the disorder is currently poorly understood, and there are no amenable animal models for the condition; maternal deficiency for the enzyme steroid sulfatase has been proposed as a potential risk mechanism. Here we show that inhibition of steroid sulfatase with 667-COUMATE (10 mg/kg p.o.) in new mouse mothers results in behavioural abnormalities that can be partially alleviated by the administration of the clinically-efficacious antipsychotic ziprasidone (0.3–1.0 mg/kg i.p.). The pattern of behavioural abnormalities in 667-COUMATE-treated mice implicated a genetic substrate at 21–23 cM on chromosome 15; of the 17 genes within this chromosomal interval, only one (*Nov*/*Ccn3*) was significantly differentially expressed in the brains of vehicle and 667-COUMATE-treated mice. Two additional members of the Ccn family (*Ccn2*/*Ctgf* and *Ccn4*/*Wisp1*) were also significantly differentially expressed between the two groups, as were three further genes co-expressed with *Nov/Ccn3* in brain (*Arhgdig*) or previously implicated in disorder risk by clinical studies (*Adcy8* and *Ccl2*). The expression of *Nov*/*Ccn3*, but not of the other differentially-expressed genes, could be normalised by ziprasidone administration (1.0 mg/kg). *NOV*/*CCN3* lies directly under a linkage peak for PP risk at 8q24, and the associated protein possesses numerous characteristics that make it an excellent candidate mediator of PP risk. Our data suggest the 667-COUMATE-treated mouse as a model for PP with some degree of face, construct, and predictive validity, and implicate a novel, and biologically-plausible, molecular risk pathway for PP.

## Highlights

• Postpartum psychosis (PP) is a severe psychiatric disorder of unknown cause.
• Steroid sulfatase (STS) deficiency may influence PP risk.
• Postpartum inhibition of STS in mice results in behavioural and genetic abnormalities.
• These abnormalities can be alleviated with an antipsychotic drug.
• The study suggests a new mouse model and a biological risk pathway for PP.

## 1 Introduction

Postpartum psychosis (PP) is a severe psychiatric disorder occurring shortly after childbirth in 1–2 out of every 1000 mothers (Sit et al., 2006). The disorder is characterised by hallucinations, delusions, cognitive disorganisation and mood problems, and is associated with an increased risk of maternal suicide or infanticide (Sit et al., 2006). The pathophysiological basis of PP is poorly understood due, in part, to the lack of an amenable animal model. The identification of biomarkers associated with increased risk is a key goal for ensuring early clinical intervention.

Increased PP risk is associated with a personal or family history of psychotic disorder (notably bipolar disorder), with precipitous drops in circulating oestrogens following childbirth, with obstetric complications including pre-eclampsia, and with psychosocial stressors (Sit et al., 2006). Small genetic linkage and association studies have implicated regions of chromosome 16p13 and 8q24 (Jones et al., 2007) and serotonergic abnormalities (Kumar et al., 2007) respectively, but have not identified robust candidate genes. Recently, immune system (Bergink et al., 2013) and tryptophan-kynurenine pathway (Veen et al., 2016) disruptions have been demonstrated in PP, whilst regular smoking is associated with reduced risk (Di Florio et al., 2015).

Maternal deficiency for the enzyme steroid sulfatase (STS) may predispose to PP (Davies, 2012); STS converts sulfated steroids to their non-sulfated forms (e.g. dehydroepiandrosterone sulfate, DHEAS, to DHEA) which may act as precursors for oestrogens and androgens (Davies, 2012). Maternal STS deficiency is associated with obstetric complications (Fernandes et al., 2010), whilst in healthy individuals, DHEAS serum levels positively correlate with postpartum psychoticism (Marrs et al., 2009); decreased postpartum DHEA levels are associated with activation of the maternal immune system (Tagawa et al., 2004).

In the first part of the study (Experiment 1), we tested whether acute inhibition of steroid sulfatase in new mouse mothers would elicit behavioural and gene expression changes of relevance to PP. STS was inhibited using 667-COUMATE (also known as STX64), a drug that systemically and irreversibly inhibits enzyme activity by >90% in rodents at the dose used here (Purohit et al., 2000). STS inhibition was indexed indirectly by measuring serum levels of DHEA(S), and levels of the stress hormone corticosterone were also measured. The behavioural tasks assayed aspects of emotional reactivity, activity and sensorimotor gating that are commonly perturbed in psychotic disorders; pup maltreatment or infanticide, which may represent a surrogate measure of PP in animals (Quilter et al., 2007) was also recorded. In Experiment 2, we tested whether 667-COUMATE-induced behavioural and gene expression changes could be reversed by administration of clinically-relevant doses of the atypical antipsychotic drug ziprasidone (Sit et al., 2006).

## 2 Materials and methods

### 2.1 Subjects and husbandry

Virgin C57BL/6JOlaHsd female mice (aged 12–26 weeks, originally obtained from Envigo UK and bred within Cardiff University School of Psychology), were housed in trios with males of the same strain. 3–5 days before giving birth, females were housed individually and were monitored closely until birth. Mice were maintained on *ad libitum* food and water, in a temperature, humidity and light-controlled room (21 ± 2 °C, 50 ± 10% humidity, lights on at 0700hr for 12 h) and were regularly inspected for signs of ill health. Experiments were performed according to the UK Animal Scientific Procedures Act (1986).

### 2.2 Drug administration

#### 2.2.1 Experiment 1

<12 h after giving birth, mothers were injected *per os* (p.o.) with either vehicle solution (tetrahydrofuran:polyethylene glycol 400: distilled water in a 1:6:3 ratio (Ireson et al., 2004)), n = 14) or 667-COUMATE (10 mg/kg, Sigma-Aldrich, UK) in the same vehicle (n = 17) in a pseudo-randomised manner. Mothers were administered the same treatment 48 h after this first injection. The 667-COUMATE administration regime was based upon previously-published pharmacokinetic data in rodents (Ireson et al., 2004, Purohit et al., 2000) and was intended to provide maximal enzyme inhibition across the postpartum period whilst minimising off-target effects. Injections were performed between 09:00–10:00 h. Behavioural testing was carried out 24 h after the second injection between 09:00–13:00 h.

#### 2.2.2 Experiment 2

<12 h after giving birth, mothers were injected *per os* (p.o.) with 667-COUMATE (10 mg/kg) as above. 24 h after the first injection, mice were injected intraperitoneally (i.p.) with either vehicle solution (1% methylcellulose in 0.9% saline) or one of two doses of ziprasidone hydrochloride (0.3 mg/kg or 1.0 mg/kg (free-base concentrations) Sigma-Aldrich, UK) in the same vehicle in a pseudo-randomised manner. 24 h later, mice received a second injection of 667-COUMATE (10 mg/kg), and 23 h after this, mice received a final injection of vehicle, 0.3 mg/kg, or 1.0 mg/kg ziprasidone. Injections were performed between 08:30–10:00h. The three experimental groups were: mice which received 667-COUMATE (10 mg/kg) with ziprasidone (0 mg/kg)(CVCV group, n = 16), mice which received 667-COUMATE (10 mg/kg) with ziprasidone (0.3 mg/kg)(CZCZ0.3 group, n = 16), and mice which received 667-COUMATE (10 mg/kg) with ziprasidone (1.0 mg/kg)(CZCZ1.0 group, n = 8). Ziprasidone doses were selected to have minimal effects on activity (Kalinichev and Dawson, 2011). Behavioural testing was carried out 1hr after the final injection between the hours of 09:30–13:00 h.

### 2.3 Homecage monitoring and behavioural analysis

Prior to injections, mother/pup health, litter sizes and weights, and maternal weights were recorded; pup deaths or signs of maternal aggression towards the pups were noted. These measures allowed us to assess whether the drug regimes were adversely affecting gross maternal and/or pup health, and/or pup maltreatment or infanticide; we were particularly concerned that inhibition of STS in the mother’s mammary gland may affect provisioning of her pups. Only mothers who gave birth to at least one live pup were included in the study.

Mothers were initially behaviourally tested on an elevated plus-maze (Isles et al., 2004) to assay anxiety-related and exploratory phenotypes. Mice were placed in a closed arm, and allowed to explore the apparatus freely for 5mins. Their lateral and vertical (‘rearing’) activity was objectively tracked using Ethovision Observer software Version 3.0.15 (Noldus Information Technology, The Netherlands); additional measures of vertical and lateral exploration (head dips from the open arms and stretch attend postures) and emotional reactivity (defecation) were recorded manually. Key measures of interest were the ratio of open arm:closed arm time and latency to first entry of the open arms (indexing anxiety), entries into the closed arms (an index of within-maze activity minimally confounded by anxiety), numbers of rears/head dips and stretch-attend postures, and numbers of fecal boli. Two seconds per animal were added to the open arm latency measure obtained from Ethovision to account for the time lag between putting the animal on the maze and initiating tracking. Following testing on the elevated plus-maze, basal activity level was tested in the dark using locomotor cages (Isles et al., 2004). The number of infra-red beam breaks made over a 1hr session (4 × 15 min bins) was recorded. Finally, sensorimotor gating was assayed using a startle and prepulse inhibition (PPI) paradigm (Dent et al., 2014); the main measures of interest were startle response over first three pulse alone trials (an index of emotional reactivity), the startle response for four levels of prepulse at 0, 4, 8 and 16 dB above background (P120, PP4P120, PP8P120 and PP16P120 respectively, an index of habituated startle and PPI), and startle response over pulse-alone trials with varying stimulus intensity (an index of auditory acuity). The behavioural tests were administered in order of increasing severity such that performance on the latter tests would not be substantially influenced by prior exposure to potential stressors. Mice were returned to their homecage for 5–10min between behavioural tests.

### 2.4 Culling and tissue collection

3 h after behavioural testing, subjects were culled by cervical dislocation. Trunk blood was collected in BD SST Microtainer Gold tubes (BD Biosciences) and serum extracted according to the manufacturer’s instructions prior to storage at −80 °C. Whole brains were removed, bisected sagitally, and frozen on dry ice.

### 2.5 Steroid hormone analysis

Serum steroid hormone levels were assayed by ELISA (dehydroepiandrosterone sulfate (DHEAS) and DHEA DRG International; corticosterone, Enzo Biosciences) according to the manufacturer’s instructions, with sample dilution as necessary and samples run in triplicate. Samples were taken from vehicle-treated mice (n = 9 for DHEA and corticosterone, and n = 10 for DHEAS), and 667-COUMATE treated mice (n = 15 for all compounds); DHEAS and DHEA levels were both measured in vehicle-treated (n = 8) and 667-COUMATE-treated (n = 15) mice. Standard curves were determined using SigmaPlot 11.0 (Systat Software Inc.) according to the equation: y = y0 + (ab/(b + x)) where y0, a and b are constants.

### 2.6 RNA extraction, cDNA synthesis and quantitative PCR analysis

High-quality total RNA was extracted from the right hemisphere of the brain using RNeasy Universal Midi Kit (Qiagen) according to the manufacturer’s instructions. For Experiment 1, RNA was extracted from vehicle-treated mice and 667-COUMATE treated mice (n = 12 per group). For Experiment 2, RNA was extracted from mice administered 667-COUMATE with ziprasidone (0 mg/kg)(n = 11), mice administered 667-COUMATE with ziprasidone (0.3 mg/kg)(n = 11) and mice administered 667-COUMATE with ziprasidone (1.0 mg/kg)(n = 6). 20 μl cDNA solution was synthesised from 4 to 5 μg RNA using RNA-to-cDNA EcoDry Premix with random primers (Clontech), and was diluted 50-fold with distilled water. Quantitative PCR analysis was performed using the ΔC_t_ method using *Gapdh*, *Hprt* and *Rn18* *s* as housekeeping genes (Trent et al., 2014) (primer sequences available in Supplemental Table S1).

Table S1

### 2.7 Statistics

Data were analysed using SPSS 20 (IBM Corporation, New York). Normal data (as determined by Shapiro-Wilk test) were analysed by unpaired two-tailed (unless stated otherwise) *t*-test/One Way ANOVA or Two Way Repeated Measures or Mixed Model ANOVA, with a between-group factor of DRUG TREATMENT and a within-group factor of TIMEPOINT or PULSE TYPE. Startle data were analysed using bodyweight and ‘pressure exerted in the absence of a stimulus’ as covariates. Where sphericity assumptions were violated in ANOVA, Greenhouse-Geisser corrected degrees of freedom values are presented. Post hoc pairwise comparisons were performed using the Sidak test. Non-parametric data were analysed by one or two-tailed Mann—Whitney *U* test. Correlational analyses for normally and non-normally distributed data were performed using Pearson test or Spearman Rank-Order Correlation respectively. P-values of <0.05 were regarded as nominally significant. Normally-distributed data are reported as mean values ± standard error of the mean, and non-parametric data as median values with 95% confidence intervals (obtained by bootstrapping).

## 3 Results

### 3.1 Experiment 1

#### 3.1.1 Litter sizes, maternal behaviours and maternal and pup health

Initial litter sizes (i.e. prior to the first injection) were comparable between the vehicle and 667-COUMATE treatment groups; the number of pups found dead was equivalently low for both groups (Table 1). All mothers built and maintained normal nests.

667-COUMATE treatment in the mother did not affect gross health or bodyweight (DRUG TREATMENT: F[1,29] = 1.18, p = 0.29, DRUG TREATMENT x TIMEPOINT: F[3,87] = 2.15, p = 0.10). Pup health was normal across the course of the experiment; mean pup weight increased equivalently across both experimental groups from ∼1.30 g to ∼1.75 g (DRUG TREATMENT: F[1,29] = 0.42, p = 0.52, TIMEPOINT: F[2.1, 61.1] = 3.39, p = 0.038, DRUG TREATMENT x TIMEPOINT: F[2.1, 61.1] = 0.47, p = 0.64).

#### 3.1.2 Elevated plus-maze

Treatment with 667-COUMATE in new mothers resulted in a significantly reduced latency to enter the open arms of the maze, and a significantly greater propensity towards rearing; these effects occurred in the absence of significant effects on other behavioural measures assessed in this test (Table 1).

#### 3.1.3 Locomotor activity

The experimental groups showed an equivalent decrease in activity over the course of the 1hr test session (from ∼515 to ∼305 infrared beam breaks per 15 min bin) (DRUG TREATMENT: F[1,29] = 0.00, p = 0.97; TIMEPOINT: F[1.68,48.7] = 24.6, p < 0.001; DRUG TREATMENT x TIMEPOINT: F[1.68,48.7] = 0.40, p = 0.64).

#### 3.1.4 Startle and prepulse inhibition (PPI)

There was no significant effect of treatment on initial mean startle response to three pulses of 120 dB (i.e. 55 dB above background)(vehicle: 55 ± 10 arbitrary startle units, 667-COUMATE: 61 ± 15 arbitrary startle units, F[1,27] = 0.01, p = 0.94).

**Fig. 1:**
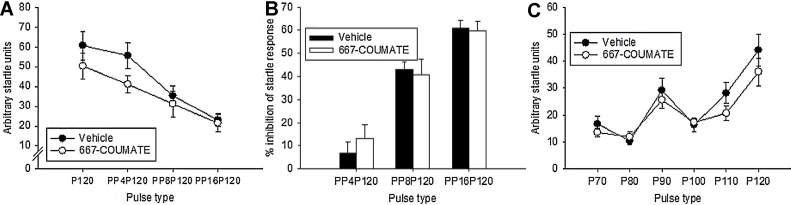
667-COUMATE-treated mice showed a reduced startle response compared to vehicle-treated mice in the absence of no prepulse stimulus (P120) or in the presence of a small prepulse stimulus 4 dB above background (PP4P120)(A). There were no significant differences between the two groups with regard to prepulse inhibition of the startle response (B), nor with startle response with varying levels of pulse intensity (70–120 dB)(C).

On the ‘pulse alone’ and ‘prepulse’ trials, there was the expected pattern of reduced startle response with increasing levels of prepulse (PULSE TYPE, F[3,29] = 54.4, p < 0.001); whilst there was no main effect of DRUG TREATMENT (F[1,29.5] = 1.41, p = 0.25), there was a significant DRUG TREATMENT x PULSE TYPE interaction (F[3,29] = 3.05, p = 0.044) with 667-COUMATE-treated mice displaying reduced startle when no, or a minimal, prepulse stimulus was present (Fig. 1A). Although there was the expected trend of increased startle inhibition with increasing volumes of the prepulse (F[1.66,48.1] = 86.5, p < 0.001), there was no significant main effect of DRUG TREATMENT (F[1,29] = 0.39, p = 0.85) nor any significant DRUG TREATMENT x PULSE TYPE interaction (F[1.66,48.1] = 0.70, p = 0.48) (Fig. 1B).

Increasing stimulus volume from background (70 dB) to 120 dB was associated with increased startle response in both groups consistent with normal hearing and motor reactivity (DRUG TREATMENT (F[1,29.2] = 1.70, p = 0.20, PULSE TYPE F[5,29] = 17.39, p < 0.001, DRUG TREATMENT x PULSE TYPE (F[5,29] = 1.68, p = 0.17)(Fig. 1C); on average, startle in drug-treated mice at pulses of 110 dB and 120 dB was lower than in vehicle-treated mice, consistent with the data presented above.

#### 3.1.5 Steroid hormone levels

Serum DHEAS levels showed the expected trend towards higher levels in the drug-treated group, whilst serum DHEA levels were, on average, lower in the drug-treated group (Table 1). For mice in which both DHEAS and DHEA levels were measured, the ratio of median DHEAS:median DHEA levels was 189 for the vehicle-treated group and 620 for the 667-COUMATE treated group (i.e. ∼3.3fold higher in drug-treated mice than in vehicle-treated mice). Consistent with our previous data in adult male MF1 mice (Trent et al., 2013), there was a significant inverse correlation between serum DHEA levels and 1hr locomotor activity in postpartum females (r_s_ = −0.416, p = 0.043). Serum corticosterone levels did not differ between groups (Table 1).

#### 3.1.6 Gene expression

**Fig. 2:**
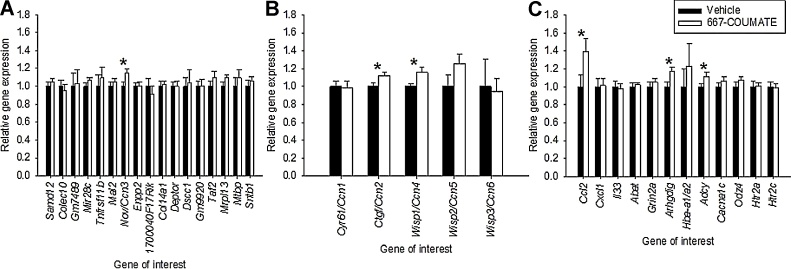
*Nov/Ccn3* was the only gene within the candidate gene interval (21–23 cM, chr 15) that was differentially expressed in vehicle and 667-COUMATE treated hemibrain (A); two further Ccn family genes (B) and three positional/functional candidate genes (C) were significantly differentially expressed between the two experimental groups.*p < 0.05.

Our behavioural data indicated drug-induced effects on elevated plus-maze rearing and latency to enter the open arms in the same task; intriguingly, these two measures are strongly influenced by a small quantitative trait locus between 21–23 cM on mouse chromosome 15 (Henderson et al., 2004), a region syntenic with human chromosome 8q24. Thus, we compared the expression of 17 genes with known products within this interval (http://www.informatics.jax.org/marker, last accessed 2nd September 2016) in vehicle and 667-COUMATE-treated hemibrains. Only one between-group comparison (for *Nov*, also known as *Ccn3*) was significant, with higher expression in drug-treated tissue (t[22] = −2.19, p = 0.039, Fig. 2A) (p ≥ 0.12 for other comparisons).

The nominally-significant effect of 667-COUMATE treatment on *Nov/Ccn3* expression would not survive correction for multiple testing. We considered that if significant effects of 667-COUMATE treatment on the expression of one or more of the other five members of the *Nov/Ccn* gene family were to be found, this would provide additional confidence that the original finding was a true effect. Both the expression of *Ctgf/Ccn2* and *Wisp1/Ccn4* were significantly increased in 667-COUMATE-treated tissue (t[22] = −2.15, p = 0.043 and t[17.27] = −2.47, p = 0.024 respectively), whilst expression levels of *Cyr61/Ccn1*, *Wisp2/Ccn5* and *Wisp3/Ccn6* did not differ between the two conditions (t[22] = 0.01, p = 0.99, p = 0.11, and t[22] = 0.67, p = 0.51 respectively)(Fig. 2B).

We then assessed the expression of three immune-related genes (*Ccl2*, *Cxcl1* and *Il33*) whose activity is thought to be regulated by *Nov/Ccn3* (Le Dreau et al., 2010, Perbal, 2006), and that have previously been implicated in PP (*Ccl2*) or bipolar disorder (*Il33*) pathogenesis (Barbosa et al., 2014, Bergink et al., 2013). Brain *Ccl2* expression levels were significantly elevated in 667-COUMATE-treated mice (t[22] = −2.28, p = 0.033), whilst *Cxcl1* and *Il33* levels were equivalent between the two groups (t[22] = −0.10, p = 0.92 and t[22] = 0.30, p = 0.77 respectively)(Fig. 2C). We also examined the expression of: a) murine orthologues of three genes suggested as positional candidates for PP (Jones et al., 2007) (*Abat* and *Grin2a* at 16p13, and *Adcy8* at 8q24), b) two genes within 16p13 whose expression correlates with *CTGF*/*CCN2* or *NOV*/*CCN3* expression in developing human brain tissue (Li et al., 2016) (*Hba-a1/a2* and *Arhgdig*), and c) two genes suggested by genomewide association studies as candidates for bipolar disorder (*Cacna1c* and *Odz4/Tenm4*)(Harrison, 2016). *Adcy8* expression was significantly higher in the brains of 667-COUMATE-treated mice (t[22] = −2.17, p = 0.041), consistent with previous evidence that adenylate cyclase (Adcy) activity might be involved in the mechanoregulation of the Ccn genes (Schild and Trueb, 2004); *Arhgdig* expression was also increased in 667-COUMATE-treated brain relative to vehicle-treated brain (t[22] = −2.375, p = 0.027)(Fig. 2C), and *Nov/Ccn3* and *Arhgdig* expression (as indexed by ΔCt values) was significantly positively correlated across both experimental groups (r = 0.51, p = 0.01). There was no effect of DRUG TREATMENT on the expression of *Abat*, *Grin2a*, *Hba-a1/a2*, *Cacna1c* or *Odz4/Tenm4* (t[22] = −0.29, p = 0.78, t[22] = −0.67, p = 0.51, t[20.2] = −1.04, p = 0.31, t[22] = −1.13, p = 0.27 and t[22] = −1.09, p = 0.29 respectively) (Fig. 2C).

Finally, we examined the expression of two serotonergic system genes: *Htr2a* (encoding a primary target for antipsychotic drug action) and *Htr2c* (the expression of which may be affected by Sts deficiency, Trent et al., 2012). The expression level of neither of these two genes was affected by DRUG TREATMENT (*Htr2a*: t[22] = 0.08, p = 0.94, and *Htr2c*: t[22] = 0.27, p = 0.79)(Fig. 2C). Overall, our gene expression data indicate a high degree of specificity to the effects of 667-COUMATE.

### 3.2 Experiment 2

#### 3.2.1 Litter sizes, maternal behaviours and maternal and pup health

Initial live litter sizes (i.e. prior to any injections) did not differ between the CVCV, CZCZ0.3 and CZCZ1.0 groups; the number of pups found dead was equivalently low for all groups (Table 2). All mothers built and maintained normal nests.

Maternal health and bodyweight was not significantly differentially affected by DRUG TREATMENT (F[2,37] = 1.83, p = 0.18, DRUG TREATMENT x TIMEPOINT: F[1.4, 76.0] = 0.34, p = 0.83). Pup health also appeared normal across the course of the experiment; mean pup weight increased equivalently across all groups (from ∼1.30 g to ∼1.80 g) (DRUG TREATMENT: F[2,37] = 0.47, p = 0.63, TIMEPOINT: F[1.5, 57.0] = 2.02, p = 0.15, DRUG TREATMENT x TIMEPOINT: F[3.1, 57.0] = 0.40, p = 0.76).

#### 3.2.2 Behaviour

We hypothesised that, in 667-COUMATE-treated mice, ziprasidone administration would attenuate rearing in the elevated plus-maze and increase latency to enter the open arms in the absence of minimal effects on activity. Overall, rearing levels were low and equivalent across the three groups (Table 2). Whilst median first open arm entry latencies did increase across the three groups, this was not significant (Table 2). The interpretation of the rearing and open arm entry data is complicated by the fact that both doses of ziprasidone tended to reduce activity in the aversive elevated plus-maze, as indexed by closed arm entries (Table 2). In contrast to the closed arm entry data in the elevated plus-maze, we found no significant difference between groups in terms of their overall number of beam breaks in the relatively non-aversive locomotor activity paradigm (Table 2), indicating that the reduced activity in the antipsychotic-treated groups in the elevated plus-maze test may have been partially due to the anxiogenic effect of ziprasidone in 667-COUMATE treated mice.

**Fig. 3:**
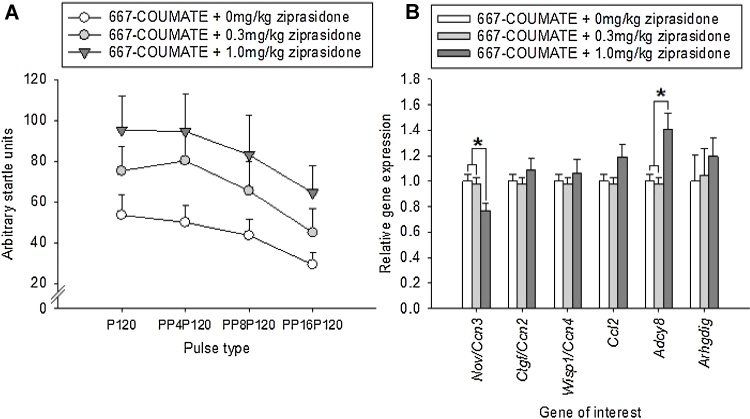
Treatment with ziprasidone dose-dependently enhanced the low startle response induced by 667-COUMATE administration (A). The highest dose of ziprasidone significantly reduced *Nov/Ccn3* gene expression and increased *Adcy8* expression in 667-COUMATE-treated mice (B). *p < 0.05.

We further hypothesised that, in 667-COUMATE-treated mice, the administration of ziprasidone would enhance the startle response, particularly when a prepulse stimulus was absent or small. Ziprasidone did dose-dependently enhance startle, though for all values of pulse-prepulse pairings (DRUG TREATMENT: F[2148] = 9.44, p < 0.001, PULSE TYPE: F[3148] = 3.29, p = 0.023, DRUG TREATMENT x PULSE TYPE: F[6148] = 0.08, p = 1.00, Fig. 3A).

#### 3.2.3 Gene expression

We compared the brain expression of genes whose expression differed significantly in Experiment 1 (i.e. *Nov/Ccn3*, *Ctgf/Ccn2*, *Wisp1/Ccn4*, *Ccl2*, *Adcy8*, and *Arhgdig* in mice treated with 667-COUMATE plus one of three doses of ziprasidone (0, 0.3 or 1.0 mg/kg)(Fig. 3B). *Nov/Ccn3* expression was significantly affected by DRUG TREATMENT, with significantly lower expression in the CZCZ1.0 group relative to the other two groups (F[2,25] = 5.07, p = 0.014, p < 0.05 for pairwise comparisons with 1.0 mg/kg group). *Adcy8* expression was significantly affected by DRUG TREATMENT, with higher expression in the CZCZ1.0 group relative to the other two groups (p < 0.01, p < 0.005 for pairwise comparisons with CVCV and CZCZ0.3 groups respectively). The expression of *Ctgf/Ccn2*, *Wisp1*/*Ccn4*, *Ccl2* and *Arhgdig* genes did not differ as a function of DRUG TREATMENT (F[2,25] = 0.90, p = 0.42, F[2,25] = 0.54, p = 0.59, F[2,25] = 3.36, p = 0.051, and p = 0.44 respectively).

## 4 Discussion

The molecular pathophysiology of PP is poorly defined, partially as a consequence of having no amenable animal models; the main animal model for PP currently is the ‘porcine infanticide model’ (Quilter et al., 2007) which, although valuable, is impractical for large-scale experimental studies and does not accurately reflect the PP phenotype (most mothers with PP are not infanticidal).

We used pharmacological techniques to produce an experimentally-amenable mouse model of PP with some degree of face, construct and predictive validity that may indicate underlying pathogenic processes. Briefly, acute postpartum inhibition of the steroid sulfatase enzyme in mouse mothers, predicted on the basis of theory to give rise to PP-related phenotypes (‘construct validity’)(Davies, 2012), elicits phenotypes of relevance to PP. In terms of behavioural face validity, 667-COUMATE-treated mice exhibited: a) a reduced startle response with preserved PPI (a phenotype seen in bipolar disorder and dependent upon levels of pro-inflammatory cytokines (Beck and Catuzzi, 2013)) and b) alterations in elevated plus-maze behaviour, likely to be influenced by a region of chromosome 15 (syntenic to the PP candidate region 8q24 in man). These behavioural phenotypes were not confounded by the effects of 667-COUMATE on maternal/pup health, or maternal activity. In terms of endocrinological face validity, 667-COUMATE treated mice, like women with PP (Epperson and Ballew, 2006), exhibited normal corticosterone/cortisol levels. In terms of molecular face validity, our model showed alterations in the brain expression of a gene previously implicated in bipolar disorder and PP (*Adcy8*) (Jones et al., 2007) and a gene encoding a pro-inflammatory cytokine (*Ccl2*) whose levels are elevated in serum of individuals with PP (Bergink et al., 2013); the abnormal expression of these genes may contribute towards the behavioural profiles of the drug-treated mice. As one 667-COUMATE-induced behavioural phenotype (reduced startle response) could be reversed by the administration of an antipsychotic used to treat PP, the model may also have some degree of predictive validity. The extent to which the model recapitulates other features of PP remains to be established. Moreover, whilst 667-COUMATE is an effective inhibitor of STS, it may have off-target effects, notably on carbonic anhydrase II (Ho et al., 2003); these off-target effects have no gross effects on maternal health, but their contribution to the more subtle changes in drug-treated mice is difficult to quantify. In support of steroid sulfatase inhibition-mediated effects on the gene expression differences reported here, DHEA influences *Ctgf*/*Ccn2* (Zhang et al., 2013) and *Ccl2* (or *MCP-1*)(Wang et al., 2011) expression and secretion in females.

Our gene expression work indicated altered expression of several members of the Ccn gene family in 667-COUMATE treated brain. This gene family, which encodes a number of secreted extracellular matrix-associated proteins expressed in the brain (Malik et al., 2015), represents an interesting candidate for effects on PP risk given: a) its known involvement in female reproductive function (Winterhager and Gellhaus, 2014), b) its dynamic brain expression throughout pregnancy and the puerperium (Ray et al., 2015), c) its modulation of Notch and Wnt signalling pathways (Winterhager and Gellhaus, 2014) that are disrupted in bipolar disorder (Pedroso et al., 2012) and cases of postpartum psychiatric disturbance (Pantoni et al., 2005) and d) the known role of extracellular-matrix dysfunction in the pathophysiology of mood disorders (Lubbers et al., 2014). Additionally, Ccn gene expression may be altered by the administration of psychotomimetic agents (Sakuma et al., 2015), by social stress (Stankiewicz et al., 2015), and by small molecules including cytokines and serotonin (Chen et al., 2014).

Using methods agnostic to gene function, we have specifically implicated *NOV*/*CCN3*, encoding a biologically-relevant protein: a) its overexpression is associated with abnormal maternal behaviour which can be reversed through antipsychotic administration (present study), b) it is located ∼138 cM on human chromosome 8, below the linkage peak indicated from a sample of individuals with bipolar affective puerperal psychosis (137.5–147.5 cM) (Jones et al., 2007), c) the associated protein is thought to modulate intracellular calcium signalling (Lombet et al., 2003) a process that goes awry in both bipolar disorder (Harrison, 2016) and PP (Riley and Watt, 1985), d) its expression is repressed by oestrogen (Vendrell et al., 2004), e) it encodes a regulator of placental angiogenesis and its expression is perturbed in cases of pre-eclampsia (Winterhager and Gellhaus, 2014), f) it is highly expressed in the cortex and limbic system of the adult human brain (Malik et al., 2015), g) it can regulate axonal outgrowth of callosal projection neurons (Park et al., 2015) consistent with corpus callosum abnormalities in PP cases (Udaya et al., 2015), h) it lies adjacent to a polymorphism associated with smoking cessation (Argos et al., 2014) and its expression is downregulated in female tissues exposed to cigarette smoke (Gueugnon et al., 2016), and i) small genomic duplications encompassing *NOV*/*CCN3* have been associated with bipolar disorder (Macayran et al., 2006). Given possible thyroid dysfunction in PP (Bergink et al., 2011), it is also interesting that cortical *Nov*/*Ccn3* and *Adcy8* expression is regulated by 3,5,3′-triiodo-l-thyronine (T3) (Berbel et al., 2014). Finally, recent work has shown that wildtype mouse mothers carrying pups with genetic mutations perturbing placental function exhibit abnormal behaviour and significantly increased hippocampal *Nov*/*Ccn3* gene expression (Creeth et al., 2015 and Hugo Creeth, pers. comm.).

The study has two main limitations. First, in the absence of data on behaviour or gene expression in non-postpartum mice administered 667-COUMATE, we cannot ascertain whether STS deficiency and its sequelae confer risk of abnormal maternal phenotypes in the postpartum period only, or confer risk of abnormal maternal behaviour though mechanisms operating both within, and outside, the postpartum period. Second, for the gene expression analyses we used a hemibrain dissection given that no individual brain region has robustly been shown to function abnormally in PP cases, and to capture large gene expression changes within one brain region or smaller gene changes throughout multiple brain regions; however, this approach may be insensitive to small gene expression changes in specific brain regions.

## 5 Conclusions

Our results indicate that acute postpartum steroid sulfatase deficiency results in abnormal maternal behaviour (at least in mice), and begin to define a brain pathway that may feasibly be disturbed in women diagnosed with PP i.e. signalling from the extracellular matrix to the cytoplasm via Ccn proteins, transmembrane proteins such as integrins and Notch, and intracellular molecules. Further details of the molecular pathway(s) by which steroid sulfatase inhibition influences postpartum behaviour may be revealed by hypothesis-free gene expression analyses in our mouse model. Our data offer support for *STS* (Xp22.3), *NOV*/*CCN3*, *WISP1/CCN4* and *ADCY8* (8q24) and *ARHGDIG* (16p13) as functional and positional candidate genes for PP risk, and indicate that analyses comparing genetic variation within these genes, or their physiological correlates (e.g. serum DHEA(S) levels), in samples of healthy nulliparous and parous controls and PP cases is warranted.

## Funding sources

The work was funded by the Wellcome Trust (105216/Z/14/Z) the Medical Research Council UK (MR/L010305/1), an Erasmus grant from University of Barcelona, and Cardiff University. The authors have no competing financial interests in relation to the work described.

## Conflict of interest statement

The authors declare no conflict of interest. The funding bodies did not play any role in the design of the study, in data collection or analysis, or in the decision to publish.

## Figures and Tables

**Table 1:** A comparison of physiological, behavioural and endocrine markers across vehicle and 667-COUMATE-treated mothers.

Measure of interest	Vehicle	667-COUMATE	Statistical significance
*Litter measures*			
Initial litter size	6.9 ± 0.4 pups	6.6 ± 0.6 pups	t[29] = 0.48, p = 0.63
Number of pups found dead per mother across course of experiment	1.5 (95%CI: 1–2)	1.0 (95%CI: 0–2)	p = 0.60

*Elevated plus maze measures*			
100x (open arm time/closed + open arm time)	25 ± 4	31 ± 4	t[29] = −1.11, p = 0.28
Latency to first open arm entry (s)	9.7 (95%CI: 3.8–14.3)	2.4 (95%CI: 2.1–4.5)	p = 0.021
Closed arm entries	24 ± 2	21 ± 2	t[29] = 1.04, p = 0.31
Rears	0 (95%CI: 0–0)	21 (95%CI: 0–81)	p = 0.040
Head dips	46 ± 9	35 ± 5	t[20.4] = 1.09, p = 0.29
Stretch-attend postures	18.5 (95%CI: 9–61)	14 (95%CI: 8–29)	p = 0.42
Fecal boli	0 (95%CI: 0–2)	1 (95%CI: 0–2.5)	p = 0.38

*Maternal serum steroid hormone measurements*			
Dehydroepiandrosterone sulfate (DHEAS, ng/ml)	4.4 (95%CI: 0.0–45.5)	32.2 (95%CI: 10.7–43.1)	p = 0.07 (one-tailed)
Dehydroepiandrosterone (DHEA, ng/ml)	0.059 (95%CI: 0.000-0.232)	0.052 (95%CI: 0.000–0.119)	p = 0.45 (one-tailed)
Corticosterone (ng/ml)	9.0 (95%CI: 5.5–13.8)	10.0 (95%CI: 3.2–19.1)	p = 0.91

**Table 2:** A comparison of physiological and behavioural markers across new mouse mothers treated with 667-COUMATE and vehicle (CVCV), with 667-COUMATE and 0.3 mg/kg ziprasidone (CZCZ0.3) or with 667-COUMATE and 1.0 mg/kg ziprasidone (CZCZ1.0). *significantly different from other two groups (p < 0.05).

Measure of interest	CVCV	CZCZ0.3	CZCZ1.0	Statistical significance
*Litter measures*				
Initial litter size	7.0 (95%CI: 6.0–8.0)	6.0 (95%CI: 5.0–7.0)	6.5 (95% CI: 6.0–8.0)	p = 0.26
Number of pups found dead per mother across course of experiment	0 (95%CI: 0–0)	0 (95%CI: 0–2)	0 (95%CI: 0–1)	p = 0.54

*Elevated plus maze measures*				
Rearing	0.5 (95%CI: 0–12.5)	2.5 (95%CI: 0–14)	0 (0–0)	p = 0.20
Latency to first open arm entry (s)	7.8 (95%CI: 4.5–15.4)	8.7 (95%CI: 5.0-12.8)	11.1 (95%CI: 6.0-26.4)	p = 0.51
Closed arm entries	28 ± 3*	20 ± 2	12 ± 3	F[2,37] = 9.44, p < 0.001

*Locomotor activity measures*				
Total infra-red beam breaks in 1hr	1837 ± 182,	1536 ± 172	1294 ± 233	F[2,37] = 1.75, p = 0.19
