# Supplementary material for: A pharmacological mouse model suggests a novel risk pathway for postpartum psychosis
Source: Psychoneuroendocrinology. 2016 Dec;74:363–70. doi: 10.1016/j.psyneuen.2016.09.019 (PMC5094271; doi:10.1016/j.psyneuen.2016.09.019)
Supplement: Table S1 [file mmc1.docx]

**Supplemental Table 1.** Primers used for quantitative PCR analyses; these were designed for high gene specificity, for optimal amplification efficacy, to amplify multiple transcripts from each gene (including protein coding transcripts), and to span exons where possible.

| **Gene** | **Forward primer (5’-3’)** | **Reverse primer (5’-3’)** |
| --- | --- | --- |
| \| *Samd12* \| \| --- \| | GCTGTCAAAACCAGTAGCCC | GCTCGCCCAGTTATGTCATG |
| \| *Colec10* \| \| --- \| | GAGTGAAAGGAGAGCTGGGT | AGATGGTACCTGCTTTGCCT |
| \| *Gm7489* \| \| --- \| | ACCACAGTCAACTCCAACCA | TCTTGGAGAGGCATCAGTGG |
| \| *Mir28c* \| \| --- \| | CACTGCAGTTACAAAGACAAATGAG | GCTGCTTGTGTTCAATAGACTGT |
| \| *Tnfrsf11b* \| \| --- \| | ATGAACAAGTGGCTGTGCTG | TCTTCCTCCTCACTGTGCAG |
| \| *Mal2* \| \| --- \| | GTACTCCGGAGCTTTCGTCT | CCTTGGAGCAGAGGTAGAGG |
| \| *Nov/Ccn3* \| \| --- \| | CCCCACAACACCAAAACCAT | TGCTTGTCTTCAGCTCCAGA |
| \| *Enpp2* \| \| --- \| | TGAGAGCCGGGACATTCTTT | CTCTCATTGTCTGGCAGGGA |
| \| *1700040F17Rik* \| \| --- \| | ATGGCTTTGACCACTCCAGA | ACTGACGACTCTTCTTGCCA |
| \| *Col14a1* \| \| --- \| | GGAAGACTTTGCCCTCCTCT | CCACGAAGGTCTCCAGAGTT |
| \| *Deptor* \| \| --- \| | AGGATGATGGGACCTTTGCA | TCTCTAGGCTGGAGGAGTGT |
| \| *Dscc1* \| \| --- \| | TCACACGGAGATCTTTGGCT | CTCCTCCTCACTTGCCTGAA |
| \| *Gm9920* \| \| --- \| | CGTTTGGATCAATGTGCCCT | CCTCCATGAGATCCAGCTGT |
| \| *Taf2* \| \| --- \| | TCAGCACAAACCTTCTGCAC | TCCCTTCAGCACCCATTCAT |
| \| *Mrpl13* \| \| --- \| | ATGTCCAGCTTCTCTAGGGC | GTCTCCACAGTCACTCAGCT |
| \| *Mtbp* \| \| --- \| | TGCAAGGTATGGGCAAGAGA | CCTTGGGAGTGACGAGAAGT |
| *Sntb1* | TCCTTTGCAACACGAACTGG | TGTGGCACCCCTGTACTATG |
| *Cyr61/Ccn1* | ACCGCGAGTTCTTTTCAACC | CACTTCACAGATCCGGGTCT |
| *Ctgf/Ccn2* | TGCCAGTGGAGTTCAAATGC | GTGTCCCTTACTTCCTGGCT |
| *Wisp1/Ccn4* | CTTCTCGGCGCTTTACTGAC | GTTTATCTGCAGTGCCTCGG |
| *Wisp2/Ccn5* | GTTTGAAGCTGGCTCCACAA | GCTGGGCATACACCATTGAG |
| *Wisp3/Ccn6* | TACTGTGACTACTCCGGGGA | TGGGTGGGGCTGAAATACTT |
| *Ccl2* | CCCTAAGGTCTTCAGCACCT | ACAGAAGTGCTTGAGGTGGT |
| *Cxcl1* | CCAGAGCTTGAAGGTGTTGC | TCTGAACCAAGGGAGCTTCA |
| *Il33* | TCCCTGTCCTGCAAGTCAAT | AGTAGTCCTTGTCGTTGGCA |
| *Abat* | GGCCACTGCTAGACTTCCTG | ACCATCAGGAACCAACAAGC |
| *Grin2a* | TTGGGAGCGGGTACATCTTT | ACAGTGTCTCCAGCTCTTCC |
| *Arhgdig* | AGATGAGGTGCTGGACGAAA | TCAGCAGTGTCAGCCTAGTC |
| *Hba-a1/a2* | GGATCCCGTCAACTTCAAGC | TGCTCACAGAGGCAAGGAAT |
| *Adcy8* | TCTGGTCTTGGGATGTGGAC | CTCTTTCCCGTGACCCTCTT |
| *Cacna1c* | ATGCCACCTTCCTGATCCAA | CAACTCCTCCTCAGCAGTCA |
| *Odz4* | TGGACGTGAAGGAGAGGAAG | AGCTGTAGGACTTCTGTGGG |
| *Htr2a* | CACCATAGCCGCTTCAACTC | AAGACTGGGATTGGCATGGA |
| *Htr2c* | CATCATGAAGATTGCCATCGTT | CGCAGGTAGTATTATTCACGAACACT |
